# Strategies for regulating tissue fibrosis and their clinical application

**DOI:** 10.1186/s41232-020-00116-9

**Published:** 2020-04-10

**Authors:** Masataka Kuwana

**Affiliations:** Department of Allergy and Rheumatology, Nippon Medical School Graduate School of Medicine, 1-1-5 Sendagi, Bunkyo-ku, Tokyo, 113-8603 Japan

Fibrotic conditions account for over 30% of deaths around the world [1]. Pathologic fibrosis can occur in virtually any organ systems, such as the liver, heart, lungs, kidneys, gastrointestinal tract, bone marrow, and skin. Tissue fibrosis is characterized by excessive accumulation of collagens and other extracellular matrix (ECM) components in parenchymal organs, replacing normal tissue architecture with stiff, acellular fibrous tissue. Fibrotic tissue remodeling is the end result of a cascade of vascular and immune responses to environmental injury and represents a failed tissue repair process, which is self-limited, resulting in scar rather than regeneration [2].

The major producers of ECM are fibroblasts, which are tissue-resident stromal cells of mesenchymal origin. Activated fibroblasts synthesize and secrete ECM macromolecules, growth factors, cytokines, and chemokines, migrate, adhere to, contract, and remodel connective tissue, and transdifferentiate into contractile myofibroblasts [3]. There is increasing recognition that fibroblasts represent a heterogeneous population of cells with diverse origins and functions, even within a single tissue. The ECM-producing cells in the tissue involve not only proliferation of resident fibroblasts, but also transdifferentiation from other tissue resident cell lineages, including pericytes, adipocytes, epithelial cells, endothelial cells, and circulating fibrocytes. ECM also plays a vital role regulating the differentiation, proliferation, migration, adhesion, biosynthetic capacity, and survival of tissue fibroblasts. Moreover, ECM serves as a reservoir for a variety of latent growth factors that regulate tissue fibrosis. Recent findings suggest that mechanical properties of the matrix microenvironment such as matrix rigidity and tension are also responsible for promoting, amplifying, and sustaining the fibrotic process [4]. In a physiologic state, ECM synthesis from fibroblasts is tightly regulated during tissue remodeling, although the impaired control of this regulating system leads to development of pathologic fibrosis. Relevant signals implicated in fibroblast activation in this context include growth factors (i.e., transforming growth factor-β, platelet-derived growth factor, and connective tissue growth factor), cytokines (i.e., IL-4, IL-6, and IL-13), chemokines (i.e., CCL2, CCL18, CXCL4, and CXCL12), vasoreactive peptides (i.e., endothelin 1 and angiotensin II), bioactive lipids (i.e., prostanoids), reactive oxygen species generated during cellular metabolism, and biochemical and mechanical signals from the surrounding ECM and via direct cell-cell interactions (i.e., Wnt, Notch/jagged, and Hedgehog signaling) [5]. Finally, chronic inflammation mediated by innate and acquired immune responses also contributes to the tissue fibrosis. A number of molecules, signals, and cell types involved in the process of tissue fibrosis are currently investigated for their potentials for therapeutic targets [6].

This thematic series focuses on updated topics on mechanisms regulating tissue fibrosis and their potential application to novel therapeutic strategies. Since mechanisms underlying pathologic fibrosis are not the same across the organ systems, this series features pathologic fibrosis in the skin, kidney, and heart, which are relevant to human intractable diseases. I hope that these review articles would offer novel insights into tissue fibrosis and contribute to development of novel therapeutic to this devastating condition.
